# Indirect health effects of the COVID-19 pandemic in Kenya: a mixed methods assessment

**DOI:** 10.1186/s12913-021-06726-4

**Published:** 2021-07-26

**Authors:** Edwine Barasa, Jacob Kazungu, Stacey Orangi, Evelyn Kabia, Morris Ogero, Kadondi Kasera

**Affiliations:** 1 Health Economics Research Unit, KEMRI-Wellcome Trust Research Programme, Nairobi, Kenya; 2 Centre for Tropical Medicine and Global Health, Nuffield department of Medicine, University of Oxford, Oxford, UK; 3 Health Services Unit, KEMRI-Wellcome Trust Research Programme, Nairobi, Kenya; 4 Ministry of Health, Nairobi, Kenya

**Keywords:** Pandemic, COVID-19, Indirect health effects, Kenya

## Abstract

**Background:**

The COVID-19 pandemic and country measures to control it can lead to negative indirect health effects. Understanding these indirect health effects is important in informing strategies to mitigate against them. This paper presents an analysis of the indirect health effects of the pandemic in Kenya.

**Methods:**

We employed a mixed-methods approach, combining the analysis of secondary quantitative data obtained from the Kenya Health Information System database (from January 2019 to November 2020) and a qualitative inquiry involving key informant interviews (*n* = 12) and document reviews. Quantitative data were analysed using an interrupted time series analysis (using March 2020 as the intervention period). Thematic analysis approach was employed to analyse qualitative data.

**Results:**

Quantitative findings show mixed findings, with statistically significant reduction in inpatient utilization, and increase in the number of sexual violence cases per OPD visit that could be attributed to COVID-19 and its mitigation measures. Key informants reported that while financing of essential health services and domestic supply chains were not affected, international supply chains, health workforce, health infrastructure, service provision, and patient access were disrupted. However, the negative effects were thought to be transient, with mitigation measures leading to a bounce back.

**Conclusion:**

Finding from this study provide some insights into the effects of the pandemic and its mitigation measures in Kenya. The analysis emphasizes the value of strategies to minimize these undesired effects, and the critical role that routine health system data can play in monitoring continuity of service delivery.

## Background

**Fig. 1:**
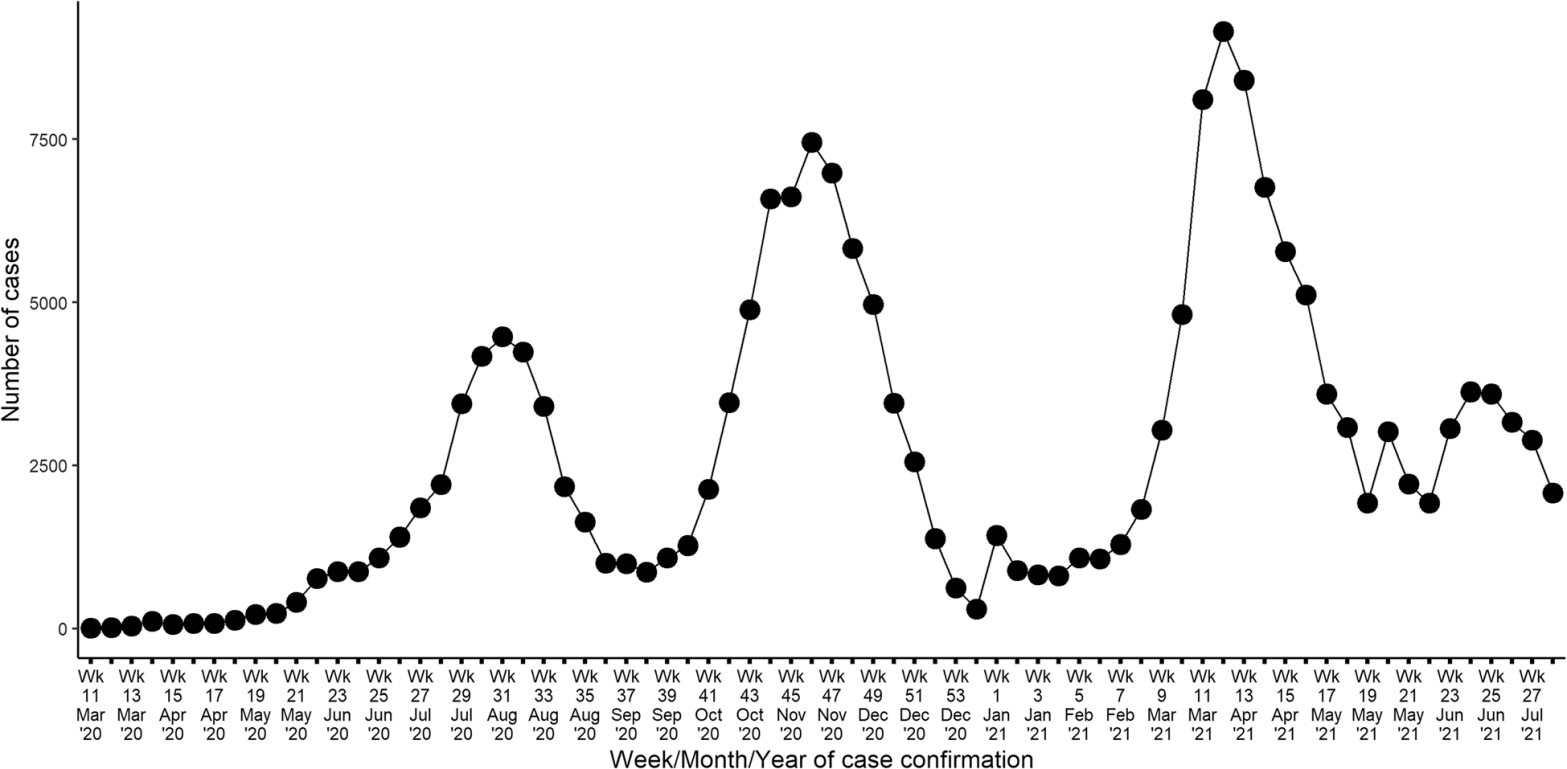
Daily incidence of COVID-19 cases in Kenya

The COVID-19 pandemic has spread to almost all countries and territories worldwide, infecting millions of individuals and causing many deaths [1]. Kenya reported its first case on 13th March 2020, and as of 31st April 2021, there were 159,318 confirmed cases and 2724 deaths [2]. Figure 1 shows Kenya’s transmission curve between March 2020 and 24th April 2021.

According to official case data, and model predictions that incorporate both case data and serology surveys [3], the country has had three waves of the pandemic, with the first peaking in July/August 2020, the second peaking in October/November 2020, and the third one peaking in March/April 2021. Compared to Europe and USA, the country’s pandemic has been characterized by 1) a high proportion of asymptomatic cases 2) a lower incidence of severe disease/hospitalizations and deaths [4].

The government of Kenya adopted several strategies to respond to the pandemic (Table 1). These include; closure of borders and a ban on international travel with an exception of cargo, closure of school/learning institutions, ban on social gatherings and meetings, a dawn to dusk curfew, closure of religious places, bars and restaurants, and observance of physical distancing (1.5 m) in spaces where people gather. The country began progressively lifting restrictions in June 2020. In addition to the above, Kenya has also relied on other non-pharmacological interventions such as testing, contact tracing, isolation and treatment, universal mandatory wearing of face masks by all in public spaces, as well as hand and cough hygiene. Kenya is among the African countries that were considered to have adopted moderate rather than highly stringent measures to balance the benefits and costs of the interventions. For instance, while other countries imposed strict lockdowns, Kenya opted for a dawn to dusk curfew and only restricted movement in counties considered epidemic hotspots.

**Table 1:** Timings and Duration of Mitigation strategies in Kenya.

Mitigation strategy	Mitigation strategy	Duration
COVID-19 Screening	Mandatory screening at all points of entry	March 2020
	Requirement for screening in all public places and buildings	March 2020
COVID-19 Testing	Testing and contact tracing	March 2020 - Ongoing
	Mandatory COVID-19 testing for truck drivers. Only those with negative tests allowed into the country	May 2020
Isolation and quarantine	14-day mandatory self-quarantine for all travelers coming into Kenya	March 2020
	Government imposes mandatory quarantine for positive patients and their contacts	March 2020
Physical distancing restrictions	Requirement for 1.5 meters physical distancing in public places	March 2020 - Ongoing
	Ban on all gatherings (including but not limited to political, social gatherings)	March 2020
	-Recommended working from home (except for employees in essential services) -State and public officers with pre-existing conditions working from home	March 2020 - Ongoing
	Regulations limiting the number of passengers in public transport vehicles	March 2020
	Restaurants remain open but a ban on opening of bars (Initially only take-aways in restaurants but lifted after 30 days)	March 2020-Ongoing
	Informal businesses remain open adhering to physical distancing measures	March 2020
	Closure of golf clubs, open sporting clubs and walking fields	April 2020
	Phased re-opening of worship places	July 2020
	Closing time for all bars and restaurants shall be 10pm every day	September 2020
	The permitted maximum size of religious gatherings is increased to one third of its normal sitting capacity	September 2020
	The permitted maximum number of persons attending funerals and weddings is reviewed upwards from 100 to 200	September 2020
	The schools in Kenya reopen from 12 October 2020 starting with examination classes, i.e. Grade 4, Class 8 and Form 4 students	12^th^ October 2020
	All political gatherings and rallies are suspended for a period of 60 days. Anyone wishing to hold such meetings to do so in town halls and limit the attendees to one- third seating capacity of the hall	November 2020
	All bars, restaurants and other establishments open to the public must close by 21h00	November 2020
Sanitation	Requirement for soap, water and hand sanitizers in public areas for hand and cough hygiene	March 2020 - Ongoing
	Mandatory wearing of face masks in public areas	April 2020 – On going
Movement restrictions	Suspension of travel for all persons coming into Kenya from any country with reported COVID-19 (except for Kenyan citizen and those with residence permits)	March 2020
	Cessation of movement into and out of Nairobi, Mombasa and Mandera (except for movement of food supplies and other cargo)	April 2020 – July 2020
	Cessation of movement into and out of Kilifi and Kwale (except for movement of food supplies and other cargo)	April 2020 – June 2020
	Cessation of movement into and out of Old town in Mombasa and Eastleigh in Nairobi	May 2020 -July 2020
	Cessation of movement persons and vehicles across the Kenya-Somalia and Tanzania International borders except for cargo vehicles.	May 2020
	Lifting of the intercountry cessation of movement in and out of Nairobi, Mombasa and Mandera effective 7 July 2020;	6^th^ July 2020
	Resumption of local air travel under strict guidelines and protocols	15^th^ July 2020
	Resumption of international air travel under strict guidelines and protocols	1^st^ August 2020
	Cessation of movement into and out of Nairobi, Kajiado, Machakos, Kiambu and Nakuru counties (except for movement of food supplies and other cargo)	April 2021
	Lifting of cessation of movement into and out of Nairobi, Kajiado, Machakos, Kiambu and Nakuru counties (except for movement of food supplies and other cargo)	May 2021
Education	Closure of all learning institutions	March 2020 - Ongoing
Curfew	Nationwide curfew – timing of curfew has been varied over time	March 2020-ongoing
Economics	State interventions to cushion Kenyans from economic shocks (tax refunds, rebates, waivers and cash transfers)	March 2020
	Launch of a National Hygiene Programme that would create jobs for the youth working in 23 informal settlements in across 7 counties	April 2020
	Government asks Nairobi City County and Kenya Power not to disconnect water and electricity over unpaid bills.	April 2020
	Allocation KES 5 billion towards local manufacture of basic medical equipment and supplies for local use and export largely by the Jua kali sector	April 2020
	Economic stimulus amounting to KES 53.7 Billion (Infrastructure, Education, Health, Small, Medium Enterprises, Agriculture, Tourism, Environment, Manufacturing)	May 2020
Workforce	Additional funds for the recruitment of additional health workers	March 2020
	Development of medical insurance package for health care workers	April 2020

Nonpharmaceutical interventions are aimed at flattening the epidemiological curve by slowing down the transmission of the virus, and hence preserving the health system capacity to meet the healthcare needs of COVID-19 patients and others, averting morbidity and mortality due to COVID-19. While these nonpharmaceutical interventions could yield positive effects such as slowing down transmission, they also result in unintended and undesired health, social, and economic effects [5]. These effects are compounded by direct effects of the pandemic. It is imperative therefore that governments monitor and mitigate the indirect effects of the COVID-19 pandemic. Existing literature on the indirect health effects of COVID-19 in Kenya has focused one or two sub-national regions (counties and one or two services For instance, Mbithi et al. [6] assessed the effect of the pandemic on HIV and TB services in one county (Nairobi), while Lagat et al. [7] assessed the effect of the pandemic on HIV testing in two counties (Homabay and Kisumu). This study aimed to assess the indirect health effects of the pandemic at a national scale, and for a broad range of service areas in Kenya. In this study, we define indirect health effects as effects of the COVID-19 pandemic on the delivery, utilization, and outcomes of routine health services (non-COVID-19 service areas).

## Methods

### Country setting

Kenya is a lower middle-income country in Eastern Africa. The country has a devolved system of governance, with a national government and 47 semi-autonomous county governments. Out of the total population, 36% lives below the poverty line [8] and 65% lives in rural areas [9]. The Kenyan healthcare delivery system is pluralistic with a 50–50% split between public and private healthcare provision. Healthcare providers are organized into four tiers, namely community, primary care, county referral and national referral hospitals. The health system is financed by revenues collected by (1) The government (national and county) through taxes and donor funding. (2) The National Hospital Insurance Fund (NHIF) through member contributions. (3) Private health insurance companies through member contributions. (4) OOP spending by citizens at points of care [10]. While communicable disease such as HIV/AIDS and Tuberculosis continue be among the leading causes of mortality and morbidity, non-communicable diseases such as diabetes and ischemic heart disease have been on the rise and feature prominently among the leading contributors to burden of disease [11]. NCDs account for 27% of the mortality and over half (50%) of total hospital admissions in Kenya [12]. For instance, mortality from cardiovascular disease is estimated between 6.1 to 8% [12], and the prevalence of hypertension and diabetes is estimated at 24.5 and 2.4% respectively [13, 14].

### Study approach

We employed a mixed methods approach to this analysis that combined the analysis of secondary quantitative data, qualitative inquiry involving key informant interviews, and document reviews.

#### Quantitative analysis of secondary data

We assessed the effect of COVID-19 restrictions on selected health service coverage and utilization indicators (Table 2). We obtained monthly data (between January 2019 and November 2020) on these indicators from the Kenya health information system (KHIS), which is the official government health management information system (HMIS) [15]. We assessed the data for missing values and outliers and adjusted for reporting rates. We replaced the outliner value using the median value.

**Table 2:** Health services indicators assessed in the study

Service/ Disease	Indicator	Numerator	Denominator
Health facility outpatient visits	Outpatient (OPD) utilisation rate	OPD attendance	Total population
Health facility inpatient admissions	Inpatient Bed Occupancy Rate (%)	Occupied bed days	Available bed days
Childhood immunization	Measles vaccination coverage (%)	Measles-Rubella doses given < 1 year	Population under 1 year
	DPT 3 coverage (%)	DPT 3 doses given < 1 year	Population under 1 year
Maternity	Percentage of deliveries conducted by Skilled birth attendants	Deliveries by a skilled health attendant	Estimated deliveries
	Four-visit ANC visit coverage	Number of pregnant women attending at least four ANC visits	Number of new ANC clients
Sexual violence	Number of sexual violence per 1000 OPD visits (> 5 years)	Total number of sexual violence cases (> 5 years)	Total OPD visits among > 5 years patients

We employed interrupted time series (ITS) analysis [16] to quantitatively evaluate the impact of COVID-19 on the level and trend of selected utilization and coverage indicators. ITS is a quasi-experimental approach for evaluating the impact of an intervention such as a policy change, community development programme, infection prevention and control initiatives and diseases such as COVID-19 by making a pre-post comparison of trends [16]. In ITS, the impact of an event is determined by assessing any change in the trend of the post-event values (observations) following an extrapolation of the pre-event trend [17–19]. We used March 2020 as the event month since it was the month where the first COVID-19 cases were first reported and control measures initiated. We conducted a single-group ITS analysis using user-written STATA command “*itsa*” specifying the *prais* model which automatically follows a first-order autoregressive [AR (1)] process that takes into account the correlation between the first-order errors [20].

The ITSA model used in these analyses was as follows [20, 21]:

$$ \mathrm{Aggregated}\ \mathrm{outcome}={\beta}_0+{\beta}_1^{\ast}\left(\mathrm{time}\ \mathrm{since}\ \mathrm{start}\ \mathrm{of}\ \mathrm{the}\ \mathrm{study}\right)+{\beta}_2^{\ast}\left(\mathrm{intervention}\ \mathrm{periods}\right)+{\beta}_3^{\ast}\left(\mathrm{time}\ \mathrm{since}\ \mathrm{start}\ \mathrm{of}\ \mathrm{study}\right)\times \left(\mathrm{intervention}\ \mathrm{periods}\right) $$

Where:

1. β_0_ is an “intercept” representing the baseline level of the outcome variable.
2. β_1_ is a “slope” indicating the trend in the outcome variable before the onset of COVID-19 and restrictions.
3. Β_2_ represents the “step-change” or the change in the outcome variable that occurs immediately following the onset of COVID-19 and restriction, which is hypothetically the effect of COVID-19 and restrictions.
4. Β_3_ is the “slope” indicating the trend post-COVID-19 and restrictions onset relative to that before the onset of COVID-19 and restrictions onset.

In these models, the time since start of study was entered as a continuous variable indicating the month since start whereas intervention periods is a dummy variable indicating pre-intervention “0”, otherwise “1” [20, 22]. These analysis were performed in STATA version 16 [23].

#### Qualitative inquiry

We conducted key informant interviews to explore COVID-19 health system disruptions that we assumed would result in disruption of service access and utilization. We assessed disruptions in the following areas:

- Financing
- Supply chain
- Health workforce
- Health infrastructure
- Service provision
- Patient access

We used a snowballing approach to identify respondents for the key informant interviews. We targeted the ministry of health (MOH) and key disease programmes and requested the program managers to identify and nominate individuals from their programmes that had information on how the COVID-19 pandemic had affected programme activities. Each programme manager nominated two individuals. We conducted key informant interviews a total of 12 ministry of health respondents. Table 3 outlines the distribution of key informant interview respondents.

**Table 3:** Key informant interview respondents

Respondent category	Number of respondents
Ministry of Health	2
National Immunization program	2
National Malaria control program	2
National HIV/AIDs control program (NASCOP)	2
National cancer control program	2
National TB control program (NLTP)	2
**Total respondents**	**12**

We audio recorded each of the interviews and later transcribed them to MS word. We analyzed qualitative data using NVIVO 12 software. We analyzed this data using a thematic approach that entailed three key steps: coding, charting, synthesis and interpretation. In the coding step, we read the transcripts to familiarize ourselves with the data and to identify emerging ideas. We then developed a coding framework that incorporated the study framework and emergent ideas from the data. We used the coding framework to code the transcripts, and subsequently chart them. We then synthesized charted data across transcripts to developed synthesized results.

## Results

### Interrupted time series results

1. Outpatient and inpatient utilization

**Fig. 2:**
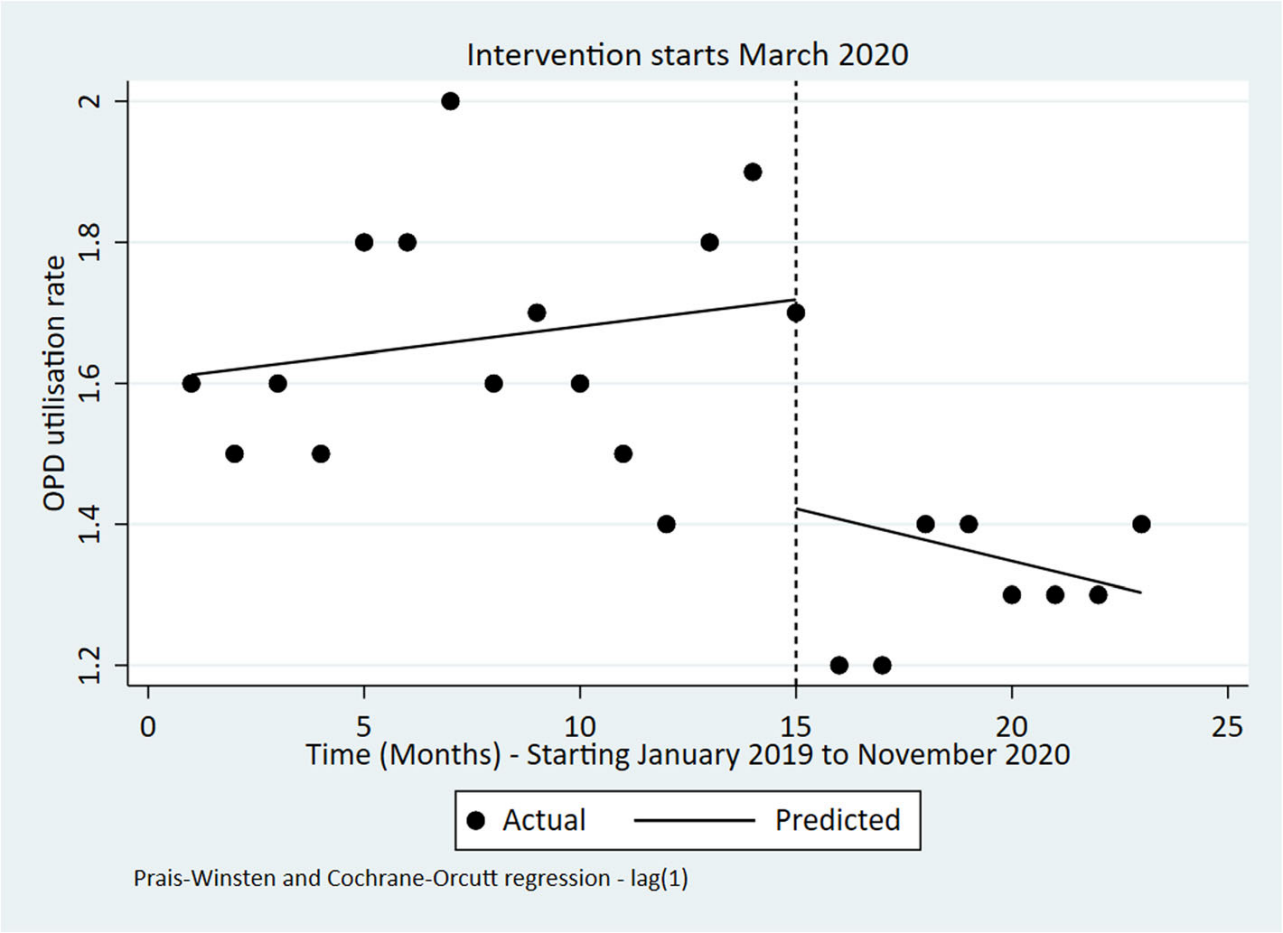
Interrupted time series of monthly OPD utilisation in Kenya from January 2019 to November 2020

**Fig. 3:**
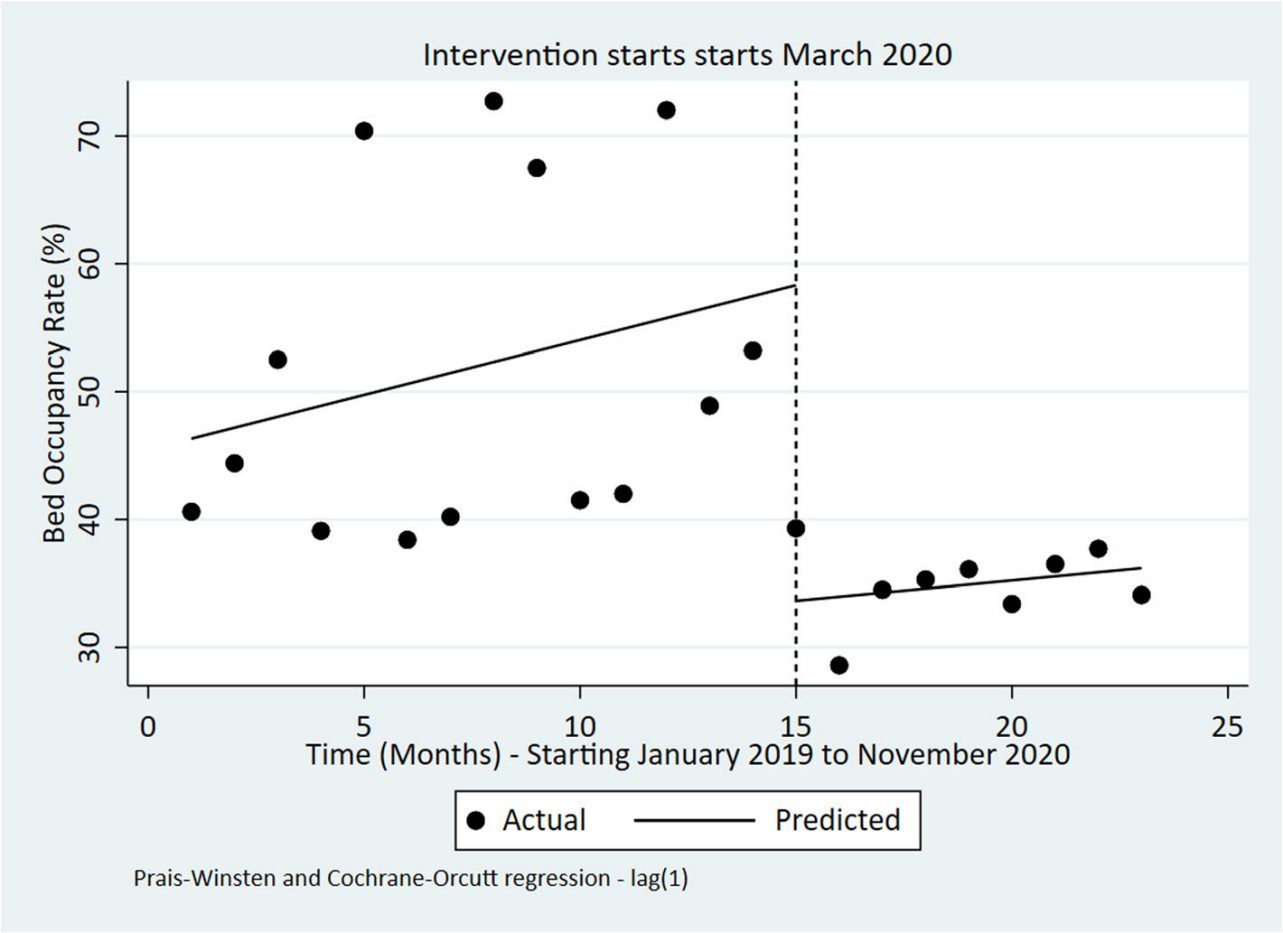
Interrupted time series of monthly bed occupancy rate in Kenya Jan 2019 to November 2020

Findings show that the outpatient utilization rate reduced in March 2020, and continued to reduce in the months after March 2020, even though all these changes in the OPD utilisation rate (before, during and after March 2020) were statistically insignificant (Fig. 2 and Table 4). On the other hand, the bed occupancy rate significantly declined by 24.67% [95% CI: − 36.15 to − 13.19; *p*-value< 0.001] during March 2020 (Fig. 3). Further, no significant change was observed post-intervention for the bed occupancy rate.

**Table 4:** ITSA outputs for each indicator

Indicator	Pre-event trend		Step change		Post-event trend (relative to pre-event trend)		Post-event Trend	
	Change [95% CI]	***P***-value	Change [95% CI]	***P***-value	Change [95% CI]	***P***-value	Change [95% CI]	***P***-value
OPD utilization rate	0.01 [−0.02 to 0.03]	0.543	− 0.30 [− 0.73 to 0.14]	0.173	− 0.02 [− 0.09 to 0.04]	0.490	− 0.01 [− 0.08 to 0.05]	0.613
Bed occupancy Rate (%)	0.86 [− 0.15 to 1.86]	0.090	−24.67 [−36.15 to −13.19]	**< 0.001**	−0.54 [− 1.68 to 0.60]	0.334	0.32 [− 0.37 to 1.00]	0.345
Number of Deliveries in health facilities	− 82 [− 854 to 691]	0.827	6002 [− 213 to 12,218]	0.058	− 206 [− 1398 to 986]	0.721	− 288 [− 1220 to 646]	0.527
Four ANC coverage visits	−0.07 [− 0.62 to 0.49]	0.807	0.15 [−5.10 to 5.39]	0.954	1.17 [0.10 to 2.24]	**0.034**	1.10 [0.29 to 1.92]	**0.01**
Measles vaccination coverage (%)	− 2.31 [−4.37 to −0.25]	0.030	44.44 [14.93 to 73.95]	**0.005**	1.54 [−3.20 to 6.28]	0.504	0.77 [−4.93 to 3.39]	0.703
DPT 3 coverage (%)	0.16 [−0.48 to 0.80]	0.609	3.14 [−4.87 to 11.15]	0.422	0.53 [−0.60 to 1.67]	0.340	0.69 [−0.21 to 1.59]	0.125
Number of sexual violence cases per OPD visits	−0.01 [− 0.02 to 0.00]	0.064	0.02 [− 0.19 to 0.23]	0.816	0.16 [0.08 to 0.23]	**< 0.001**	0.15 [0.07 to 0.22]	**0.001**

### Maternal health services

**Fig. 4:**
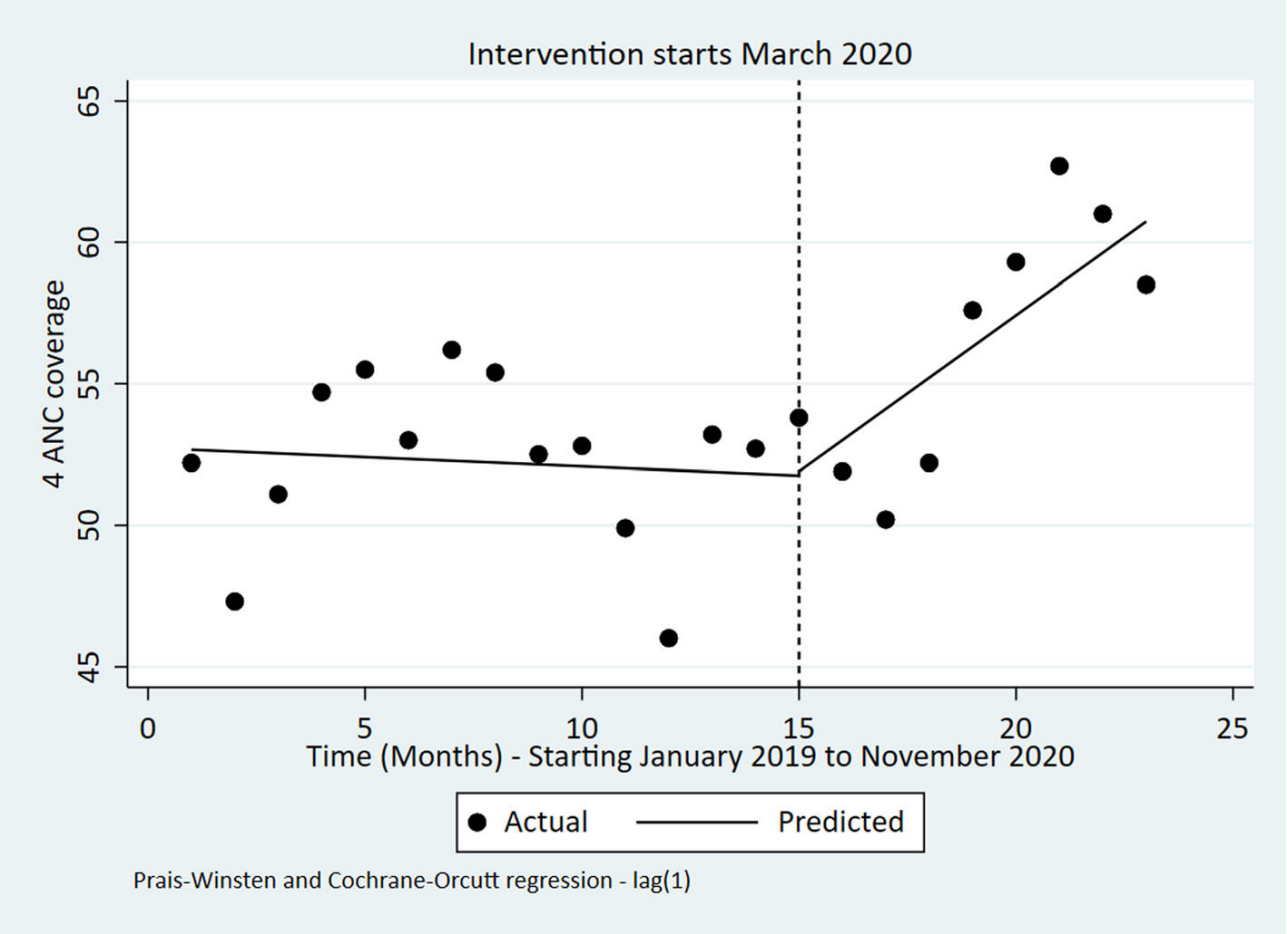
Interrupted time series of 4 ANC coverage in Kenya from January 2019 to November 2020

**Fig. 5:**
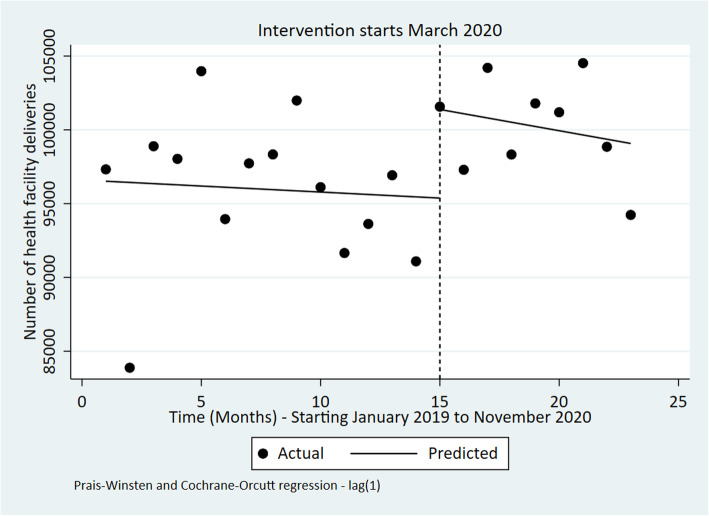
Interrupted time series of monthly number of deliveries in health facilities in Kenya from January 2019 to November 2020

The ITS analysis shows that both four ANC coverage and health facility deliveries increased in March 2020 and the months that followed even though these changes were not statistically significant Figs. 4 & 5 and Table 4.

### Childhood vaccinations

**Fig. 6:**
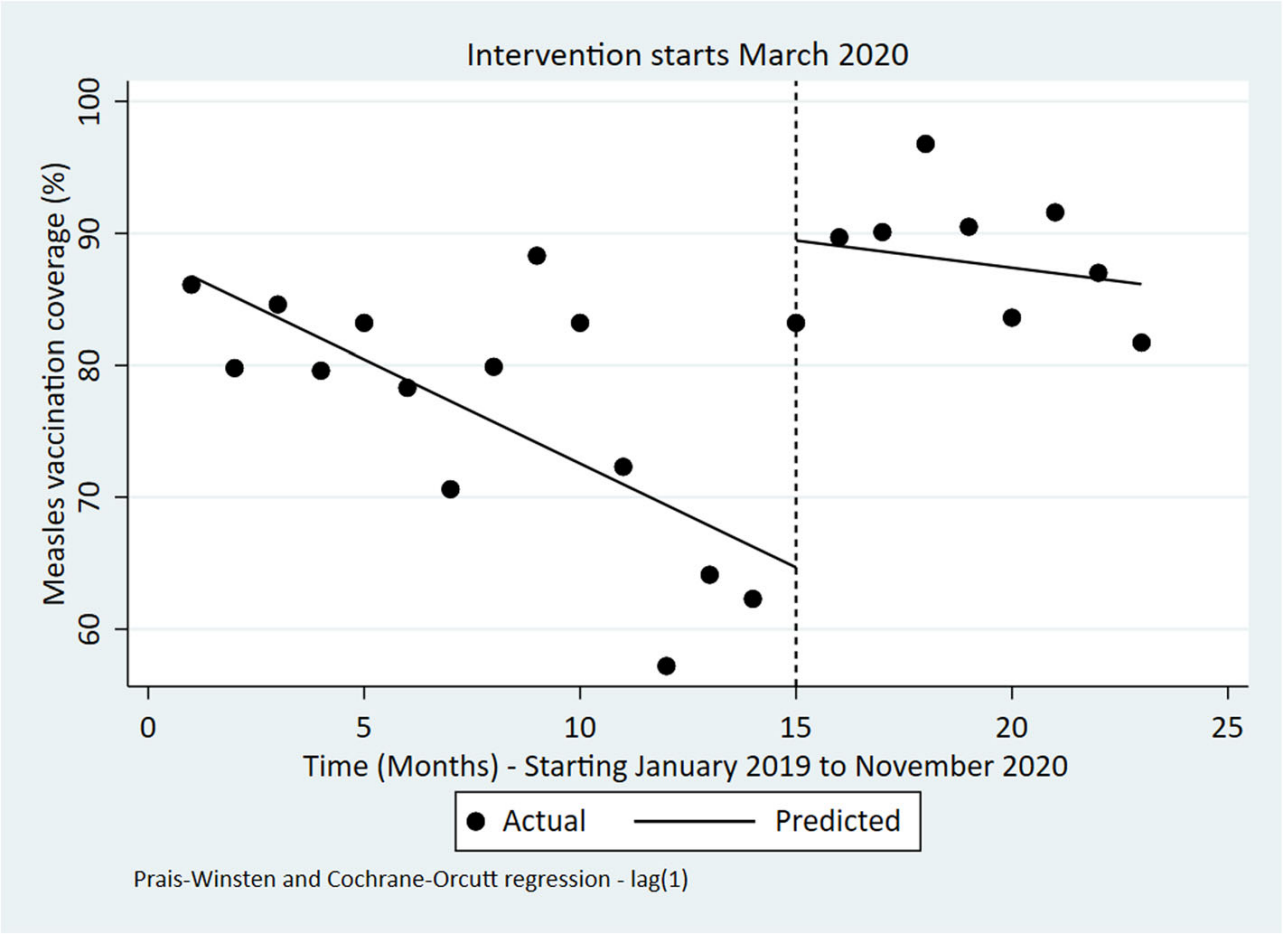
Interrupted time series of monthly Measles vaccination coverage in Kenya Jan 2019 to November 2020

The results show that there was a significant declining trend in measles vaccination coverage at an average of 2% ([− 4.37 to − 0.25], *p*-value = 0.03) per month before March 2020 (Fig. 6). However, a significant step increase in measles vaccination coverage associated with COVID-19 was observed during the intervention where measles vaccination coverage increased by 44.44% [95% CI: 14.93 to 73.95; *p*-value = 0.005]. After March 2020, measles vaccination coverage decreased monthly at a rate of 0.77% even though this was not statistically significant.

**Fig. 7:**
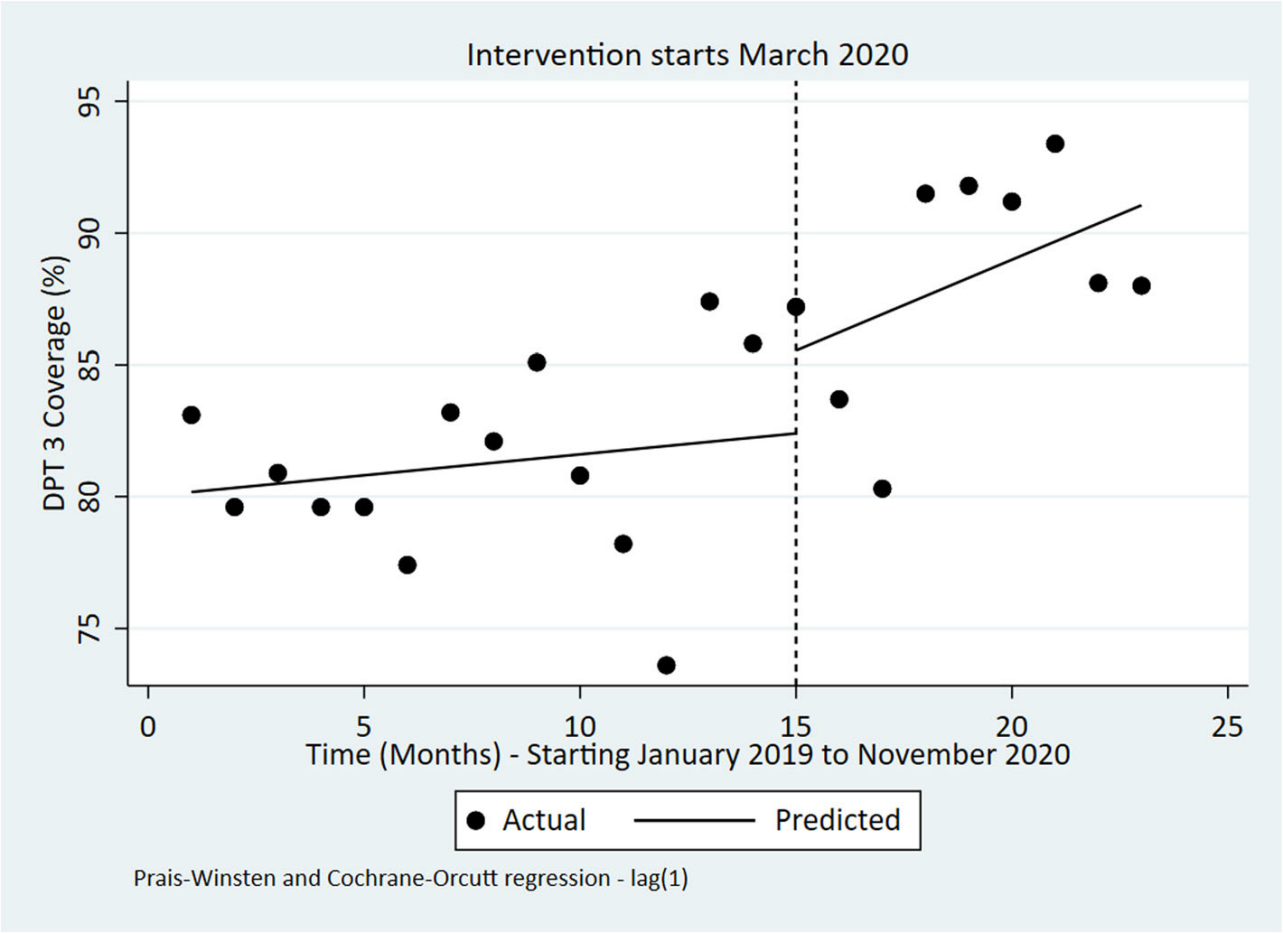
Interrupted time series of monthly DPT 3 vaccination coverage in Kenya Jan 2019 to November 2020

Unlike the trend in measles coverage, DPT 3 coverage seemed to increase before, during and after the intervention although these average changes were not significant (Table 4 and Fig. 7).

### Number of sexual violence cases per OPD visit

**Fig. 8:**
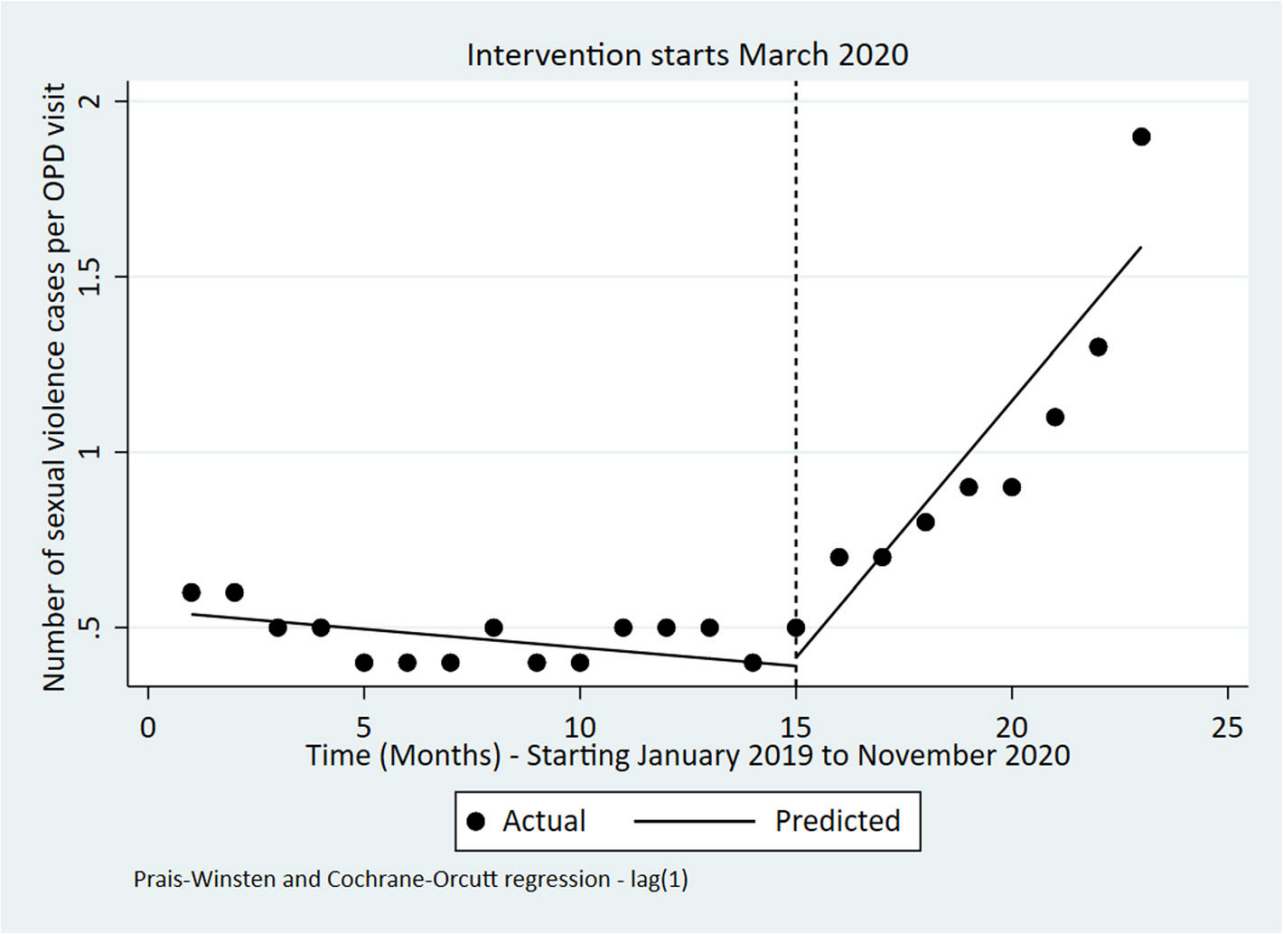
Interrupted time series of sexual violence cases per OPD visits in Kenya from January 2019 to November 2020

Overall, sexual violence cases per outpatient visit increased over the period January 2019 to November 2020. For instance, the postintervention trend, relative to the preintervention trend, indicated that the number of sexual violence cases was increasing by a factor of 0.16 cases (*p*-value< 0.001) per outpatient visit. Further, sexual violence cases increased at a monthly rate of 0.15 cases after March 2020 (post-intervention period) (Table 4 and Fig. 8).

### Qualitative results

#### Funding of essential health services

Funding for essential health services appears not to have been substantially affected by COVID-19 and government response measures in the short term. It was reported that domestic budgets for health were not reallocated.

*“We cannot say that COVID-19 and country response has really affected the domestic funding for malaria. We did not get any budget cuts”* *Malaria program staff*.

Even though there was some reallocation and re-prioritization of funding by donors, it was reported that this did not affect service delivery since the reallocation was done on savings, and the funds were replenished. For instance, the Global Fund requested the MOH to reallocate USD 5,000,000 to the COVID-19 response. However, these reallocated funds were identified from savings from planned activities that were no longer feasible due to COVID-19 measures such as face to face trainings of health workers. Further, the Global Fund provided additional funds to the country.

*“This year we [the malaria programme] realized savings of USD 6 million … USD 5 million was allocated for malaria services but the USD 1 million was reallocated to COVID-19”* *Malaria program staff*.

*“I have not seen any budget cuts for our HIV programme because of COVID-19. I think part of the savings that we had on our global fund grant was used to support COVID-19. We have not been affected.”* *HIV programme Staff*.

#### Supply chain for essential health commodities

Overall, local supply chains for essential health commodities were not affected. This includes the distribution of health commodities from the central medical stores (Kenya Medical Supplies Agency] to healthcare facilities. This was because the government had put in place measures to facilitate the procurement and distribution of essential health commodities to healthcare facilities. For example, the TB program had adequate 6 months stock at the central stores and TB facilities had a 3-month stock buffer. Further, movement to distribute essential health commodities was exempted from the movement restriction imposed as part of the country response to COVID-19.

*“At the start of the pandemic, we [TB programme] had adequate stock at the Kenya Medical Supplies Agency [KEMSA] and in healthcare facilities. This is because the policy is that we keep a 6-month stock level at the central stores and 3 months at facility level. We haven’t had a stock out for most of the commodities and if we did, it had nothing to do with COVID-19.” TB programme staff.*

*“We [the national vaccines programme] supplied sufficient doses of vaccines to health facilities in February and March 2020. This mitigated against COVID-19’s disruption of supplies and delivery of vaccines.” Vaccines programme staff.*

However, international supply chains i.e. the importation of health commodities for local use were substantially affected by global supply chain disruptions because of closure of international borders and air travel and lockdowns in source countries.

*“We [malaria programme] had supply chain challenges for commodities that we procure overseas, especially India and parts of China as well as US. We had challenges in ensuring that the commodities, especially RDTs and malaria, were delivered in time”* *Malaria program staff*.

*“We [TB programme] experienced some delay in the delivery of some Gene-XPERT cartridges. These cartridges were delivered in September because most suppliers could not deliver on time because of the COVID 19 movement restrictions.” TB programme staff.*

*“We got complaints from the oncologists about the challenges they were having accessing some drugs because factories in their source countries had been shut.”* Cancer program staff.

The cancer control program faced challenges with supply of personal protective equipment because the procurement for PPE was re-prioritized for COVID-19. PPE’s are required for the preparation and administration of cancer chemotherapy.

*“The N-95 masks that we [Cancer control programme] traditionally use for chemotherapy preparation suddenly became on demand for COVID-19. They [KEMSA] told us that priority will be given to COVID-19”* *Cancer program staff*.

The MOH employed several strategies to mitigate the negative impacts of supply chain disruptions. These included leveraging on a network of development partners to supply commodities whose supply had been disrupted and rationalizing and redistribution of existing supply of commodities.

*“The [malaria] program reached out to various development partners to provide support on mitigating the impacts of COVID-19. These included the Global Fund, the WHO and others. We did not get to a point where we were stocked out at the facility level”* Malaria program staff.

*“We [the cancer programme] realized that we might not get any supplies for PPEs to our 10 chemotherapy centers. We therefore reached out to some development partners to get us PPEs which were quickly distributed to these 10 centers”* *Cancer program staff*.

There was also active monitoring using supply chain information systems and virtual platforms such as social media to ensure that stocks levels were adequate at any given time.

*“We [TB programme] use so many platforms including WhatsApp. There’s a WhatsApp group for the field staff where they can tell us what they’re lacking in a particular area and this allows us to take action.” TB program staff.*

#### Impact on infrastructure

Country response to COVID-19 affected the availability of healthcare infrastructure for essential health services. Some facilities that were used to deliver essential services were designated as COVID-19 isolation facilities. These included treatment sites and storage facilities. This for instance affected the management of drug-resistant TB or TB patients that had challenges with adherence to therapy since therapy for these patients were typically monitored at the facility (directly observed treatment).

*“We have a policy on isolation of people who have poor adherence to TB medicines to have them in a supervised environment, especially multi-drug resistance TB patients. Some of our [TB programme] isolation centres were converted to COVID-19 isolation centres, affecting our ability to isolate and supervise these TB patients” TB programme staff.*

*“We [Vaccines programme] had some disruption of vaccination centers in facilities that were designated as COVID-19 isolation centers. We had to move vaccination services to neighboring healthcare facilities” Vaccine programme staff.*

Some laboratory infrastructure were also assigned to provide diagnostic services for COVID-19. For instance, molecular testing platforms for cancer diagnostics were assigned to COVID-19 testing. Some diagnostic machines become unavailable for purchase by the cancer program because were prioritized for COVID-19 testing.

“*COVI-19 testing is done on the molecular testing platform. We [cancer control programme] had a few challenges accessing test kits for cancer tests that are normally conducted on the molecular testing platform like the HPV test.”* *Cancer program staff*.

*“At that time of COVID-19, we [cancer control programme] were trying to secure a machine for cancer diagnostics. However, we were told that the same machine is used for COVID-19. Imagine you plan to start a diagnostic service and then COVID-19 happens and you are told you cannot purchase the machine because COVID-19 is now the priority”* *Cancer program staff*.

Measures taken to adapt to the infrastructure challenges included transferring patients to alternative facilities that were nearby, transitioning some care services such as direct observation of TB patients to home-based care.

*“We [HIV programme] transferred the clients out to the nearest facility which provides HIV services. Clients are now able to access services from that facility. There is no client who has been denied drugs because your facility has been closed.” HIV programme staff.*

To reduce the impact of increased uptake of molecular testing platforms for COVID-19 testing, patients were prescribed for alternative tests that did not require the use of molecular testing platforms.

#### Impact on health workforce

The health workforce for essential services was impacted in several ways. First there was fear of infection with COVID-19 among health workers especially because of inadequate supply of personal protective equipment (PPE). Respondents reported that this fear led health workers in lower level health facilities to refer patients who presented with fever to high level health facilities and hence increasing the workload for health workers at these higher-level facilities. Second, health workers such as laboratory officers, programme coordinators and disease surveillance officers were redeployed to focus on COVID-19.

*“Part of the staff who manage malaria services, such as disease surveillance officers, malaria coordinators, and laboratory officers were redeployed to support the COVID-19 response. This included the management of the quarantine centers”* *Malaria program staff*.

Third, COVID-19 physical distancing requirements meant that fewer patients were attended to at any given time which required health workers to extend working hours late into the night to enable them attend to all the patients.

Several measures were adopted to mitigate this effect on health workforce. These included procuring and distribution of PPE’s to health workers, the development of protocols to guide health workers on working during the pandemic, and training health workers on infection, prevention and control. The MOH also implemented mental health programs that addressed health worker burnout.

#### Impact on service delivery

The health system experienced service delivery disruptions because of the covid-19 control measures. For instance, the malaria programme has not been able to carry out some activities including the campaign for mass LLIN. Case identification and notifications for TB reduced because of a reduction in the number of patients that came to health facilities. HPV vaccination has been disrupted because a key delivery platform, schools, was shut down as part of the COVID-19 mitigation measures. Further, activities that required health workers to reach out to communities were also affected by movement restrictions. These included for instance TB contact tracing, outreach camps for cancer, and cancer screening. Postponement of elective surgeries had a negative impact on cancer patients who required surgery.

*“Some key interventions and activities, including the LLIN campaign, were either delayed. So, for example the world malaria day was canceled, the Kenya malaria indicator survey was postponed, and the mass nets campaign was also postponed”* *Malaria program staff*.

*“We [TB programme] noticed a decrease in achievement of our targets. Case notification went down … so that means we missed putting a sizeable number of the population on TB treatment. This can have an impact downstream in terms of transmission.”* *TB programme staff*.

“*There was directive that elective surgeries be deferred and that really affected patients who were scheduled for surgery because majority of surgeries for cancer are not emergencies. There was a lot of delay and the more we delay the more the disease advances and hence the harder it is to treat but this has since been revised*” *Cancer program staff*.

Adaptations to service disruptions included the use of virtual platforms to plan for delayed activities. Drug collection schedules for chronic care patients was revised to a longer period and measures were put in place to allow patients who were already on treatment to continue collecting their drugs. Where this was not possible, arrangements were made for health workers to deliver medicines to people’s homes.

*“*We [TB programme] immediately responded by revising the schedule for drug collection. *We also did a lot of technical assistance by visiting counties and providing support to optimise case detection and discuss strategies on how to bring back the case detection to optimum levels. We also try as much possible to be online and provide online support by WhatsApp.”* *TB programme staff*.

Further, health workers were issued with letters that authorized them to travel anytime despite the travel ban and the curfew.

#### Impact on patient access

Respondents also felt that patient access to services has been affected in several ways. There was a decline in the number of patients that visited health facilities because of movement restrictions and the fear of getting infected with COVID-19. Further, COVID-19 related movement restrictions prevented patients from accessing specialized cancer services that are not available locally e.g. bone marrow transplants. Vulnerable populations were especially affected. For instance, the elderly faced a higher risk of contracting COVID-19 while visiting healthcare facilities. The cancer program for instance advised elderly cancer patients to send their caregivers to collect their medication on their behalf and provided guidance on how the elderly should be protected if they needed to go to the clinic.

*“In counties like Nairobi and Mombasa where we had lockdowns, the number of patients accessing cancer treatment significantly dropped because of the lockdown. Patients were not really able to access services that easily.”* *Cancer program official*.

However, because of measures put by government to mitigate against these impacts, access and utilization was only affected for a short period of time, and utilization bounced back quickly.

*“I think the mitigation measures put by the MOH worked because when I look at the data I see that most of our services were seriously affected within the month of April, but by May things started to pick up.”* *HIV programme staff*.

Mitigation measures employed by the ministry of health included the use of community health workers to deliver some health services, such as malaria case management to individuals in their homes.

“*We [malaria program] supported community healthcare workers to carry out what we call community case management of malaria. This entailed community health workers visiting households and providing care to patients. This reduced the disruption of service access to these patients”* *Malaria program staff*.

While HPV vaccination was affected because of closure of schools, other vaccines, such as measles were not affected because the disruption occurred around the time that the MOH was carrying out a catch-up exercise to make up for vaccine stock outs earlier in the year.

*“We [vaccines programme] had a stock-out of measles vaccines between November 2019 and January 2020. When we got stocks, we distributed them to health facilities and asked them to make up for the period of stock outs by reaching out to unvaccinated children. This explains the increase in measles vaccination in March 2020”* *Vaccines programme staff*.

The use of telemedicine was also encouraged for consultations and use of virtual platforms to engage with patients, caregivers and healthcare workers. However, patients who are not economically well off had challenges using these platforms.

*“We [cancer programme] encouraged tele-medicine. We encouraged doctors to call their patients who had not been the facilities for a long time. We also encouraged patients to call their doctors if they experienced symptoms. We also had webinars for patients, caregivers and healthcare workers”* *Cancer program staff*.

## Discussion

This study set out to explore the indirect health effects of the COVID-19 pandemic in Kenya. Several observations emerge. First, there is evidence that some services were disrupted by the COVID-19 pandemic and the measures put in place by the government to contain it. For instance, the utilization of both outpatient and inpatient healthcare services reduced in March 2020, even though only the reduction in inpatient admissions was found to be statistically significant. These finding corroborates findings from other settings as shown, for instance, by the World Health Organization (WHO) pulse surveys of 105 countries in 2020 [24, 25] and country specific analyses in Uganda [26], South Africa [27], and the Philippines [28]. However, again consistent with pulse surveys, not all services were disrupted. The analysis did not show any disruptions in health facility delivery, ANC visits and measles and DPT3 vaccination coverage. Rather, the utilization of these services increased, even though this increase was not statistically significant except for measles vaccination coverage. The increase in measles vaccination is however unrelated to COVID-19 and is attributed to a planned catch-up campaign to make up for vaccine supply shortages in preceding months. The seemly minimum disruption of essential services in Kenya could be explained by the fact that Kenya’s restrictions were moderate rather than severe; curfews provide for more flexibility compared to hard lockdowns. It may be the case that these moderate restrictions minimized unintended effects.

Second, our analysis found that while some services were disrupted at the start of the pandemic, utilization of these services bounced back in the follow-up months. Similar findings have been reported in Uganda [26]. The second round of the pulse survey also reported a reduction in service disruption across the 105 surveyed countries, compared to the level of disruption reported in the first survey [25]. This could be as a result of mitigation measures put in place by the Kenya MOH and county governments. Key informant interviewees reported several measures taken by the MOH and counties to preserve the delivery of core health services that included issuing guidelines for continuity of essential services, exempting healthcare workers and individuals faced with emergencies from movement restrictions, the use of community health workers to deliver essential services to households in need, the promotion of digital technologies, and the revision of drug collection schedules for chronic care patients. This is corroborated by several MOH guidelines on the continuation of core health services during the core pandemic [29]. Further, COVID-19 restrictions in Kenya were in place for a short time period and accompanied by measures to mitigate the unintended effects on the health system. It may be the case that these mitigation measures worked.

Third, our analysis reported a statistically significant increase in sexual violence cases in the months after the introduction of COVID-19 restrictions. The increase in sexual violence cases corroborates media reports and other analysis that sexual and gender-based violence cases increased during the COVID-19 pandemic as a result of physical distancing measures that required people to stay at home. For instance, respondents taking part in a nationally representative survey reported a 37, 29, and 22% increase in domestic violence against women, men, and children respectively in their locality since the imposition of the dusk to dawn curfew in Kenya [30]. Further, the national sexual and gender based violence hotline recorded an increase in reports of gender-based violence from 86 cases in February 2020 to 1108 in June 2020 [31]. These findings also mirror findings in other countries including Uganda [26] South Africa [27], Morocco [32], India [33], and Bangladesh [34].

Fourth, key informant interviews revealed disruptions in health system functions and initiatives by the MOH to mitigate the indirect impacts of the pandemic on the health system. While financing and local supply chains appear not to have been affected, international supply chains were disrupted and affected health care services that relied on imported commodities. Human resource for health was affected in two ways. First, was the reallocation of staff to the COVID-19 response. Second, and perhaps more substantial, is the concerns by health workers of the risk of infection because of a scarcity of PPE’s. This corroborates media reports of health worker discontent with the MOH’s effort to protect them by availing PPE’s leading to health workers strikes to demand for, among others, adequate PPE’s [35]. Other disruptions experienced include the reallocation of health infrastructure to the COVID-19 response by for instance converting health facilities to COVID-19 isolation centers, and the disruption of service delivery activities such as the distribution of LLINs and access to services because of physical distancing measures. The impact of these disruptions may however have been mitigated by government measures such as stock-pilling of supplies, dispensing enough medicines to patients with chronic diseases to last longer durations, and the use of community health workers to deliver some health services.

Overall, it appears that indirect health effects of the pandemic have been minimal, unlike the indirect socio-economic effects. For instance, regarding economic impact, the country’s GDP growth rate projection for 2020 was revised to 1% down from 5 to 6% [36], while an estimated 1.7 million Kenyans lost their jobs due to COVID-19 between April and June 2020 [37]. The unemployment rate for populations aged 15–64 years doubled (10.4%) between April and June 2020 compared to that reported in the January and March 2020 (5.2%) with youths aged 20–24 and 25–29-years accounting for the highest proportion of the unemployed and the highest increase in unemployment (> 10%) [37]. Food security has also been affected. For example, a survey of residents of low-income areas in Nairobi showed that 94% had reduced their spending on food while 42% feared suffering from hunger in the future if the pandemic continued [38].

This study has several limitations. First, the quantitative analysis of changes in the level of service utilization is prone to bias from several sources including well document data quality issues of HMIS data, the likely impact of the pandemic on information systems (e.g. disruptions in reporting), and the lack of a control. Second, the qualitative inquiry is limited by a small sample size that is unlikely to have achieved saturation. Third, the qualitative inquiry only targeted national level MOH officials. Extending the sample to include frontline healthcare providers at the local level would have provided a more comprehensive view of experiences. These weaknesses notwithstanding, the data presented provide a glimpse of the likely early effects of the pandemic on the health system and provides a foundation for further, more key informant analysis once more data becomes available.

## Conclusion

COVID-19 has indirect health impacts in Kenya. These effects are however mixed, with some services affected more than others. This analysis emphasizes the value of strategies to minimize these undesired effects and to protect the delivery of essential health services. The analysis also highlights the value of routine data in monitoring continuity of service delivery and informing decisions about where interventions should be targeted to preserve health system functioning. Improving the quality and timeliness of routine health system data is therefore a critical intervention that could aid country response to epidemics.

## Data Availability

The DHIS data used this study is publicly available in the following link https://hiskenya.org/dhis-web-commons/security/login.action

## Abbreviations

PPE: Personal protective equipment
MOH: Ministry of health
WHO: World health organization
HIV: Human immunodeficiency syndrome
TB: Tuberculosis
NLTP: National leprosy and tuberculosis programme
NASCOP: National AIDS and sexually transmitted diseases control programme
LLIN: Long lasting insecticidal nets
KEMSA: Kenya medical supplies agency
ANC: Antenatal care
ITS: Interrupted time series
OPD: Outpatient department
DPT: Diphtheria, pertussis, tetanus
